# Invasive Ureteral Aspergillosis Causing an Abscess: A Report of a Rare Case

**DOI:** 10.7759/cureus.67650

**Published:** 2024-08-24

**Authors:** Saravanan Jambunathan, Senthil Kumar T, Balaji Subramaniam, Shantanu Chandrashekhar, Saravanan K

**Affiliations:** 1 Urology, SRM Medical College Hospital and Research Centre, Chennai, IND

**Keywords:** liposomal amphotericin b, invasive aspergillosis, immunocompromised hosts, ureteral aspergillosis, ureterectomy, double-j stent, voriconazole therapy, ureteric stricture, feeding jejunostomy

## Abstract

Aspergillosis is a vicious fungal infection that can develop in immunosuppressed patients. The presence of aspergillosis in the ureter or elsewhere in the genito-urinary tract is highly uncommon and rarely reported in the literature. Here, we present a 54-year-old gentleman, with uncontrolled diabetes, who presented with urosepsis. Right hydronephrosis and ureteric stricture with urinary extravasation were seen on imaging. Right percutaneous nephrostomy was done, with drainage and analysis of the pus revealing the growth of Aspergillus fumigatus species. On open exploration, a ureteric abscess cavity, which was adherent to the duodenum, was drained and uretero-ureterostomy along with feeding jejunostomy was performed. Histopathological examination and special staining confirmed the growth of aspergillosis. The patient was treated with antifungal agents and responded well with an uneventful post-operative recovery.

## Introduction

Aspergillosis is a vicious fungal infection that is known to develop in immunosuppressed patients. Commonly occurring in lungs, the presence of aspergillosis in the ureter or elsewhere in the genito-urinary tract is uncommon and rarely reported in the literature [1]. The diagnosis and treatment of the disease are challenging owing to the poor concentration of antifungal agents in the urine [2]. Prompt recognition and an early initiation of therapy can reduce the associated morbidity and mortality drastically. We present a case of a 54-year-old male with isolated invasive aspergillosis of the ureter along with urosepsis and ureteric obstruction. Despite a turbulent course of treatment, the patient responded well after urinary diversion, supported by adequate antifungal therapy.

## Case presentation

A 54-year-old gentleman, with uncontrolled diabetes mellitus for 25 years, presented with fever and right loin pain for five days. He had an associated history of passing turbid and foul-smelling urine. No history of hematuria or other urinary symptoms was noted. No other systemic illnesses were present. He had a history of left percutaneous nephrolithotripsy for left renal calculus five years back as well as a history of spine stabilization surgery done one year back. Later, he was admitted twice with urosepsis with bilateral hydroureteronephrosis for which he underwent bilateral ureteroscopy which showed extensive papillary necrosis on both sides. Hence, he was taken up twice for bilateral ureteric stenting followed by subsequent stent removals 6 months back. On examination, the patient was found to be febrile with severe tenderness over the right loin.

**Figure 1:**
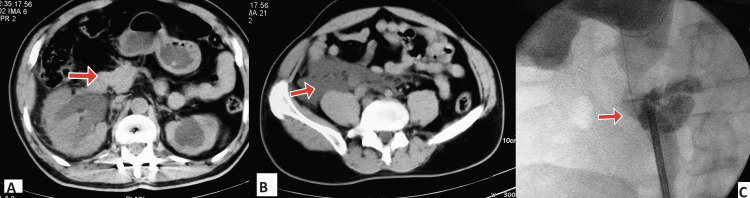
Computed tomography images with axial cuts showing (A) right hydronephrosis with (B) urinary extravasation. (C) Retrograde pyelogram (antero-posterior view) showing extravasation of contrast, as marked by the arrows.

Blood investigations revealed a rise in the total leucocyte count of 18,200 cells/cu mm (normal range: 5,000-11,000 cells/cu mm), due to the prevailing infection and inflammation. His creatinine was found to be 3.2 mg/dl (normal range: 0.7 - 1.2 mg/dl) attributing to an obstructive uropathy. Urine culture revealed the growth of Aspergillus fumigatus species. Ultrasound and non-contrast computed tomography (CT) of the abdomen were done and showed right hydroureteronephrosis and urine extravasation in the upper ureter as shown in Figures 1A, 1B.

**Figure 2:**
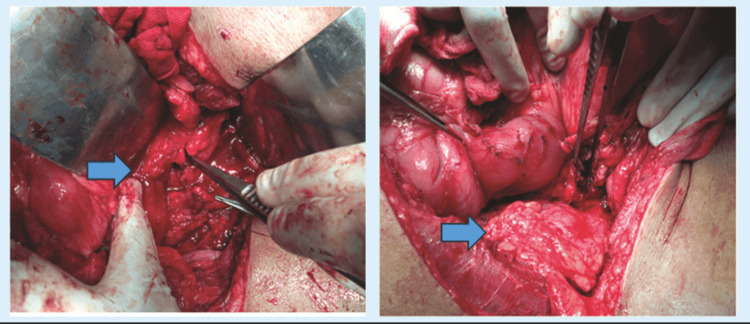
Intraoperative images showing intra-abdominal (A) ureteral abscess cavity adherent with duodenum and (B) Omental wrapping around the anastomosis site and intact dissected duodenum, as marked by the arrows.

He was subjected to right percutaneous nephrostomy (PCN) and retrograde pyelogram, which showed contrast extravasation in the lower ureter, as shown in Figure 1C. Purulent drainage of around 2000 ml/day was noted from the PCN while the patient was passing clear and adequate amount of urine via the urethra. Urine culture showed growth of fungal elements. Hence, the patient was kept under an antifungal cover of intravenous amphotericin. Post drainage, the serum creatinine dropped from 3.7 mg/dl to 1.3 mg/dl. The patient was then evaluated and taken for open exploration of the right ureter. A dilated upper ureter with a lower half stricture leading to an abscess cavity was noted, which was adherent to the wall of the second part of the duodenum. Hence, the stricture segment of the ureter was resected and the abscess cavity was drained followed by uretero-ureterostomy and feeding jejunostomy as shown in Figure 2. A double J (DJ) ureteral stent was placed across the anastomosis site.

**Figure 3:**
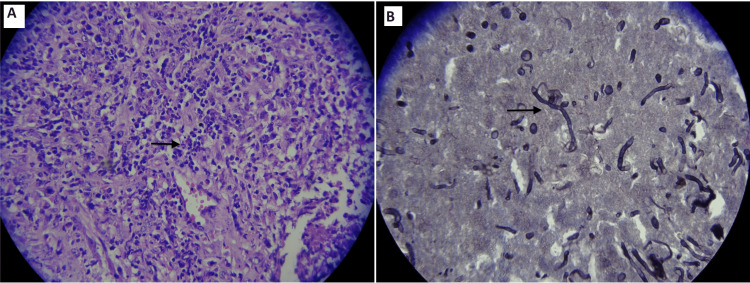
(A) Histopathological examination showing few isolated hyphae-like structures with narrow septae and acute angular branching. (B) Gomori methanamine silver (GMS) stain detecting Aspergillus fungal hyphae (100x, GMS stain).

The histopathological examination (HPE) report showed an acute or chronic inflammation in the ureteric stricture segment. The abscess cavity was found to show necrosis with mixed inflammation and fungal elements suggestive of invasive aspergillosis. Culture revealed the growth of Aspergillus fumigatus, which was resistant to fluconazole. Gomori methanamine silver (GMS) stain, the special stain for detecting fungal elements, was found to be positive as well, as shown in Figure 3.

Postoperatively, the patient had an uneventful stay. After the HPE report, the patient was maintained on oral voriconazole 200mg twice daily for three months. After six weeks, urine culture revealed no growth after which DJ stent removal was done. The patient improved significantly during the follow-up period.

## Discussion

Aspergillus is a filamentous fungus isolated from air, soil, decaying vegetables, or dust. The most common variants of Aspergillus involved are Aspergillus fumigatus and Aspergillus flavus. Mortality associated with systemic aspergillus infection is around 40 to 50%, while the mortality associated with genitourinary aspergillosis is shown to be around 28% [1,3]. Genitourinary infection is highly uncommon and usually involves hematogenous spread, ascending infection, or obstructive uropathy. Infection by aspergillosis occurs more commonly among patients with immunocompromised status such as diabetes mellitus, malignancy, and post-organ transplantation status [4-6]. Ureteral aspergillosis can commonly present with fever, flank pain, nausea, vomiting, hematuria, or dysuria along with nephromegaly or obstruction of the ureters, which may even lead to death [7,8]. The diagnosis is confirmed by the isolation of the fungus from the tissue or fluid. The recommended treatment is treatment with antifungal agents, with intravenous amphotericin B being the most effective drug along with oral fluconazole. Among all the azoles, only fluconazole has the unique property of urinary excretion while the other drugs such as voriconazole and posaconazole, are metabolized in the liver, with poor urinary excretion [4,9]. Treatment of a fungal ball can be benefited by an excision prior to antifungal therapy. This can be achieved via open or endoscopic antegrade or retrograde approaches [10,11]. Usage of isolated medical therapy in cases of associated urinary obstruction is not proven to be beneficial, needing surgical bypass of the obstruction [2,9,12]. In diabetics, achieving simultaneous sugar control plays an important role in controlling the infection.

Our patient presented with urosepsis due to ureteric obstruction, which was initially controlled with urinary diversion and intravenous amphotericin B and antibiotics. This was essential in controlling the sepsis. On further surgical exploration, the obstruction was found to be due to an abscess cavity arising from the lower ureter causing a stricture. Urine culture was found to be positive for the growth of Aspergillus fumigatus, which was resistant to fluconazole. Complete abscess drainage along with excision of the stricture under the cover of intravenous antibiotics and oral voriconazole was a key factor in having an uneventful postoperative recovery. There have been attempts to treat localized genitourinary aspergillosis with local irrigation of amphotericin B, which has seen little success. Although controversial, it should be kept among the various treatment options available in the treatment of localized disease [13].

## Conclusions

Whenever a patient with immunosuppression and sepsis presents, aspergillosis of the genito-urinary tract is considered an important part of the differential diagnosis. An early initiation of treatment along with an appropriate urological intervention can speed up the course of recovery, reducing the associated high morbidity and mortality rate.
